# Effects of a self-care program on quality of life of cirrhotic patients referring to Tehran Hepatitis Center

**DOI:** 10.1186/1477-7525-3-35

**Published:** 2005-05-18

**Authors:** Mitra Zandi, Mohsen Adib-Hajbagheri, Robabeh Memarian, Anooshiravan Kazem Nejhad, Seyed Moayed Alavian

**Affiliations:** 1 Kashan University of Medical Sciences and Health Services, kashan, Iran; 2 Tarbiat Modarres University, Tehran, Iran; 3 Baghiyatallah University of Medical Sciences, Tehran Hepatitis Center, Tehran, Iran

## Abstract

**Background:**

Chronic liver disease especially liver cirrhosis is one of the medical problems that substantially reduces the quality of life of its victims. Because of the chronic and irreversible nature of the disease, it needs self-care programs to be developed according to client's needs and to maintain their independence and sense of well-being. The purpose of this study was to determine the effects of a self-care educational program on Quality of Life (QoL) of a sample of Iranian cirrhotic patients.

**Methods:**

A quasi-experimental study was conducted on 44 cirrhotic patients in Tehran Hepatitis Center. Longitudinal case registry and random allocation technique were used to divide the sample into experimental (n = 21) and control (n = 23) groups. Chronic liver disease questionnaire (CLDQ) was used for measuring the quality of life. The experimental group was given a questionnaire to assess their educational needs. A self-care educational program was conducted and the patients were followed for 3 months. Then the quality of life of both groups was compared using descriptive and analytical statistics.

**Results:**

The experimental and control groups were the same concerning the effective factors on the quality of life, such as age, sex, etc (P > 0.05). There was no significant difference between QOL mean score of both groups before the intervention, however the QoL significantly improved in the experimental group after the intervention (P= 0.001), while the QoL decreased in control group.

**Conclusion:**

The result of the present study confirmed the positive effects of the educational and self care programs on the QoL of cirrhotic patients. Extensive educational and self-care programs along with long-term follow up such as the program conducted in this study are suggested.

## Background

Cirrhosis represents a late stage of progressive hepatic fibrosis characterized by distortion of the hepatic architecture and the formation of regenerative nodules. It is generally considered to be irreversible in its advanced stages [1,2].

While alcohol abuse is the most common cause of cirrhosis in the western world, hepatitis B is the primary cause in the third world [1]. The only relatively acceptable remedy for this condition is liver transplantation, currently performed with many limitations in Iran because of complex operation technique and high expenses [3].

Cirrhosis of the liver is the third leading cause of death in people between the ages of 25 and 65 years, exceeded only by cardiovascular disease and cancer. Cirrhosis and chronic liver diseases accounted for more than 25,000 death and 373,000 hospital discharges annually in the adult in the United States. The cost of cirrhosis in terms of human suffering, financial burden, and loss of productive life is devastating [2,4,5].

Complications such as encephalopathy, ascites, bacterial peritonitis, and frequent bleeding from variceal veins dramatically alter the well being of cirrhotic patients [6] as well as their Quality of life. Studies have shown the negative effects of the disease on the patients' activities, social functioning, and emotional status [7-10]. Because of irreversible nature of the disease, and because the current therapies are not yet good and available enough, patients quality of life has moved to the forefront of patient concern.

Several studies have documented impaired health-related quality of life (HRQoL) associated with chronic hepatitis [11,12]. In 1998, Foster et al compared the Health Related QoL (HRQoL) of liver patients with viral hepatitis B and C and reported that social functioning, energy and fatigue and role limitations due to physical problems were significantly more impaired in hepatitis C patients [14]. Younossi et al found an increasing impairment of generic HRQoL with increasing disease severity; while Marchesini et al found that the most relevant determinants of impaired health status were severity of disease and muscle cramps [7,8,15].

These studies contributed substantially to our knowledge of the physical, social and mental problems of chronic liver patients. However, little is published related to the QoL of these chronic liver patients in Iran. Moreover, none of these studies included the effect of self-care programs on the QoL of these patients, while Quality of life research as well as self-care researches could contribute to fulfill the quest for providing a better living for these chronic patients. According to Orem, self-care is a learned behavior, which can satisfy many needs of patients, provide growth and development, and prevent deviation from health [16]. So, The purpose of this study was to determine the effects of a self-care program on QoL of a sample of Iranian cirrhotic patients.

## Methods

This quasi-experimental study was conducted with two experiment (n = 21) and control (n = 23) groups in Tehran Hepatitis Center (THC) in 2002. Using a longitudinal case registry method, 44 cirrhotic patients were selected over a four months period. Age between 20 and 65, not having a coexisting chronic debilitating illness [i.e. diabetes, chronic renal failure (CRF), stroke, inflammatory bowel disease, epilepsy and malignancy] and preferably living in Tehran) were considered as inclusion criteria. Three hundred cirrhotic patients were under the THC at the time of study. The patients had a routine visit each six months that would change to a monthly visit if any problem occurred. A total of 49 cirrhotic patients were referred for their routine (monthly or six-monthly) visit during the (first 4 months of the) study period. Of these, 44 patients had the including criteria and 5 patients were excluded because they had a coexisting chronic illness (4 because of diabetes and 1 for CRF). Random allocation technique was used to divide the sample into the experimental and control groups. To do this the patients were allocated into the groups as every other one.

### Instruments

Four instruments were used including a demographic data questionnaire (consisting of questions related to the age, sex, level of education, martial status, number of children, occupation, frequency of hospitalizations, etiology, the duration and severity of the disease), a need assessment questionnaire, a self-assessment questionnaire, and the CLDQ questionnaire.

### The need assessment questionnaire

Need assessment form was consistent of a list of 20 pre written questions related to the common problems such as fatigue, itching, dry mouth, muscular cramps flatulence, and also common problems related to dietary regiment and drug therapy. Each patient wanted to respond to the questions as "yes" or "no". At the end of need assessment forms the patients were asked to determine their own preferred time for attending the educational programs.

### The self-report questionnaire

Six self-care checklists were designed in accordance with the six common problems and the content of educational programs (i.e. nutrition, controlling worry and depression, mouth dryness, pruritus, fatigue, muscular cramps). Each checklist was tabled for 30 days and included a list of self-care activities related to a common problem. The patients were asked to do a daily review on the checklists and mark self-care activities they followed. All the checklists were given to all members of experimental group and they were instructed how to complete them. They were also instructed to return the checklists to the researcher at the end of each month and new self-care checklists were given to them for the new month (a sample checklist is presented as additional file [see additional file 1]).

### QOL instrument

CLDQ: The CLDQ is the first liver specific instrument for measuring QoL in chronic liver disease (CLD) developed by Younossi et al. [6]. The CLDQ includes 29 items in the following domains: abdominal symptoms, fatigue, systemic symptoms, activity, emotional function and worry. It has 7 likert scale type of answers ranging from "all of the time" to "none of the time" (possible range 29–203 from worst to best QoL) [6]. The construct validity of the CLDQ was supported by a strong correlation with patients' global rating scores (*r *= 0.84; p = 0.02) [6,8]. A particular strength of the CLDQ is that all phases of the validation process included patients with both hepatocellular and cholestatic liver disease of varying severity (no cirrhosis to Child's C cirrhosis). This should allow the CLDQ wide application in hepatology research.

### Translation of CLDQ

The original version of CLDQ was translated into Farsi according to the standardized guidelines proposed by Guillemin et al. [17]. A native English speaker living in Iran who understood Farsi language quite well and did not have knowledge about QoL carried out back translation. The final version derived from reconciliation of the original and back translation and tested on 5 patients with chronic liver diseases. The content validity of translated CLDQ was approved by 10 faculty members in Tehran medical university. Translated CLDQ also was repeated in 5 patients in 2 weeks apart for test-retest analysis. Reliability was determined from Cronbach's alpha and test-retest. Cronbach's alpha was higher than 0.91 for domains and it was 0.93 for overall scores. Spearman's rank correlation was also 0.89 for CLDQ and it was higher than 0.73 for CLDQ domains.

### The procedure

At the first stage, all subjects were administered CLDQ and demographic questionnaire. The experimental group was additionally given the need assessment questionnaire to determine their own educational needs as well as their own preferred time for attending the educational program. The severity of the illness was diagnosed and documented by a physician in patients' records.

Then the patients' demographic data and their educational needs were analyzed. Although the educational needs were somehow different for different patients, however, all of them were expressed their interest for intending the full educational program.

The content of educational program was designed [in six domains] based on the content of need assessment questionnaire and the domains of CLDQ questionnaire. [These were consisted of the nature of disease, coping strategies in systemic symptoms, coping strategies in worry and depression, relaxation techniques, diet and nutrition, and medical therapies, its side effects and relieving factors].

The experimental group was divided into four small groups with regard to their levels of education and their free time. The content of educational program were similar in all groups except for its simplicity [based on the subjects educational level]. Four educational sessions were held for each group. Each session lasted for 45 minutes and 5–6 patients with 3–4 relatives took part in each session [the relatives played a supportive role to maintain and restore clients' independence as much as possible].

The educational sessions consisted of a talk delivered by the main researcher. Also there was scope for questions and discussions. Posters, slides and manikin were also used to facilitate the participants learning process. Educational pamphlets or handouts (totally, 5 pamphlets and 2 handouts) covering all the contents and a related self-care checklist were given to the patients at the end of each session. The content of the four educational sessions are summarized in Table 2.

**Table 2:** The content of four educational sessions

Session 1	Nature of the disease	Etiology, transmission route, clinical manifestations, diagnosis, management and complications
Session 2	Coping strategies in systemic symptoms	Fatigue, dry mouth and, pruritus
Session 3	Coping strategies in worry, nutrition	Anxiety, relaxation techniques, diet and nutrition,
Session 4	Muscular cramps, Medical therapies	Medical regimen and its side effects, and relieving factors

The self-assessment checklists were given to the patients after the fourth educational session. The patients wanted to do a daily review on the checklists and mark each self-care activity if they followed them. Checklists were similar for all patients and did not modified during the study. The patients followed the program for 3 months and recorded the interventions in the self-report checklists. The main researcher phoned patients every two weeks and checked them for their compliance for the educations and reinforced them for completing the self-report forms. The completed checklists were returned to the researcher in a monthly visit at the end of each month and new self-care checklists were given to them for the upcoming month. After follow-up for three months, all patients (including the controls and experimental groups) completed the CLDQ once again. Then the CLDQ was analyzed and the two groups were compared. This study received ethics approval from the ethic committee of Tarbiat Modarres University. All subjects provided written consent before participation. The control group was also given the educational materials at the end of the third month to observe ethics. Data analysis was performed by SPSS using descriptive and analytical statistics.

## Results

One patient in the experimental group and three in the control group died during the study to change the number of subjects in each group into 20. Demographic data of both groups are presented and compared in Table 1. No significant difference was found between the two groups.

**Table 1:** Demographic data of cirrhotic patients in the control and experimental groups

**Items**	**Control**	**Experimental**	**P**
**Mean age and SD**	46 + 12.5	40 + 12.5	0.18
**Sex (No.), %**			
Male	(14), 70	(10), 50	0.19
Female	(6), 30	(10), 50	
**Education**			
Illiterate	(2), 10	(6), 30	0.27
Primary or secondary school	(6), 30	(4), 20	
Diploma or higher	(12), 60	(10), 50	
**Marital status**			
Single	(2), 10	(2), 10	0.38
Married	(16), 80	(17), 85	
Divorced	(2), 10	(1), 5	
**No. of Children**			
Less than or equal to 3	(14), 70	(11), 55	0.32
More than 3	(6), 30	(9), 45	
**Occupation**			
Laborer	(6), 30	(8), 40	0.32
Employee	(4), 20	(6), 30	
Housewife	(4), 20	(4), 20	
Student	(2), 10	(2), 10	
Retired	(4), 20	-	
**Duration of illness (Years)**			
1–3	(12), 60	(14), 70	0.32
3–6	(6), 30	(4), 20	
More than 6	(2), 10	(2), 10	
**No. of hospitalizations**			
None	(12), 60	(8), 40	0.41
1	(2), 10	(4), 20	
3	(6), 30	(8), 40	
**Etiologic factor**			
Hepatitis B	(10), 50	(9), 45	0.83
Hepatitis C	(2), 15	(4), 20	
Autoimmune disease	(4), 20	(4), 20	
Cryptogenic	(4), 20	(4), 20	
**Child of cirrhosis**			
A	(4), 20	(4), 20	0.77
B	(8), 40	(10), 50	
C	(8), 40	(6), 30	

In general, 14 relatives took part in educational sessions with a range between 30 to 50 years of age and mostly between 40 and 50. Thirteen relatives were female, most of them had high school diploma (6 persons) and the majority were the spouses of patients (9 persons).

Patients' educational needs were used as a basis for planning educational sessions. The most common educational needs included: controlling or reducing abdominal distention (the greatest need, %70); curative ways in cirrhosis (being treatable or not treatable); ways of controlling fatigue (%65); principles of care and proper medications (%60); worry (%55); controlling pruritus and fatigue (%50); ways to decrease muscular cramps, dry mouth, and dyspnea; patterns of activity, rest, and sleep; routes of transmission as well as diagnostic tests (%45); and diagnostic procedures (%40).

In general, the items that the patients marked in the checklists were appraised over 3 months. All patients marked the nutritional items, of which %95 were observed. Items for fatigue, anxiety, and depression were ticked in %60 of cases and the rest reported no problem in this regard. Items for muscular cramps, dry mouth, and pruritus were ticked in %40, %30, and %20 of cases respectively and the rest reported no related problem.

**Figure 1:**
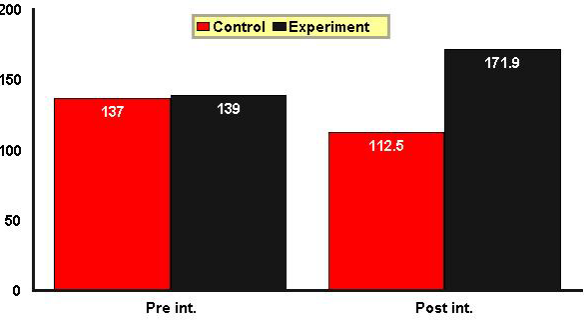
Mean score of CLDQ in the control and experimental groups before and after intervention

Figure 1 shows the total score of CLDQ in both groups before and after the intervention. Mean score of CLDQ in the control group before the intervention was 137 and changed to 112.2 after 3 months, which showed a marked decline and a significant difference by Wilcoxon test (P = 0.001). In the experimental group, the mean score was 139 and changed to 171.6, which showed a rise and a significant difference by the same test (P = 0.001). There was no significant difference between QoL scores of both groups before the intervention, which was verified by Mann-Whitney test (P = 0.75). In fact, there was no significant difference before the intervention in total score of QoL between the two groups, but the same test showed a significant difference (P = 0.001), which indicated an improvement in QoL of the experimental group after 3 months.

**Figure 2:**
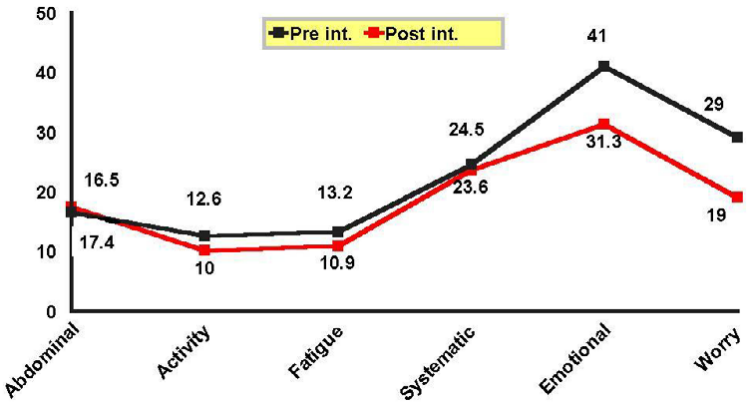
Mean score of CLDQ domains in cirrhotic patients in the control group before and after intervention

Each domain in the CLDQ was compared before and after intervention in both groups. As presented by the Figure 2, there was a significant difference between mean scores of activity (P = 0.001), worry (P = 0.001), and emotional (P = 0.001) domains in the control group before and after intervention with a decline after 3 months. In other words, subjects in the control group experienced more emotional, anxiety, and activity problems, but no significant difference was shown in systemic (P = 0.59) and abdominal (P = 0.39) symptoms as well as fatigue (P = 0.9) after 3 months (Figure 2).

**Figure 3:**
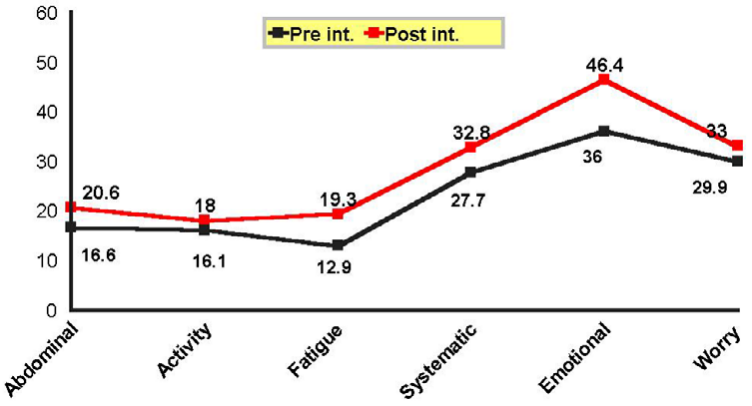
Mean score of CLDQ domains in cirrhotic patients in the experimental group before and after the intervention

On the other hands, a significant difference noted between mean scores of the abdominal symptoms (P = 0.001), fatigue (P = 0.001), systemic symptoms (P = 0.001), activity (P = 0.01), worry (P = 0.001) and emotional (P = 0.001) domains in the experimental group before and after the intervention with an overall increase than before (Figure 3). Accordingly, subjects in this group experienced less abdominal, systemic, emotional, fatigue, and worry problems as well as improved activity.

There was a significant difference between the experimental and control groups in all CLDQ domains after the intervention, as confirmed by Mann-Whitney test (P = 0.001). In other words, subjects in the experimental group experienced less systemic, abdominal, emotional, and fatigue problems than the control group. Thus, research hypothesis, "QoL of cirrhotic patients will improve following the self-care program," was verified.

However, the Wilcoxon test showed no significant difference in the severity of the disease before and after the intervention in control (P = 0.057) and experimental (P = 0.66) groups. In other word, the intervention had no effect on the severity of the disease. Mann-Whitney test also showed no significant difference in both groups before and after the intervention (P = 0.73).

## Discussion

According to our knowledge this was the first study that evaluated the effects of an educational/ self-care program on the QoL of cirrhotic patients. The results of this study confirmed the beneficial effects of educational and self-care programs on the health related quality of life and also supported the findings of previous studies that reported improvement in patients QoL after self-care and educational programs [18]. This is an important finding since cirrhotic patients showed a significantly worse disease-specific and generic HRQoL than non-cirrhotic patients or healthy controls [19].

Most patients in our study were males with age between 20–50. The most common etiologic factors for their cirrhosis were also hepatitis B, cryptogenic cirrhosis, autoimmune hepatitis, and hepatitis C. These findings were similar to the previous study conducted by Alavian et al [3].

Medical practitioners have traditionally focused on organic diseases and their treatment. Patients, however, are concerned with their symptoms, regardless of the presence of organic or non-organic findings. On the other word, the quality of life is the forefront of patient concern [20,21]. Since current therapies are not yet good enough to eradicate the disease, the patients' care should considerably focus on their quality of life [21].

Coping strategies and the ways for controlling or reducing symptoms like abdominal distention; fatigue and pruritus were among the most common educational needs among our patients. These findings indicate that our health care providers including the medical practitioners and nursing staff do not exert enough effort for the patient education. This was also evident in the low level of QoL of both experimental and control groups before the intervention that was lower than the scores of QoL in healthy people reported by the other researchers [7,8,22]. So more attention should be paid to this important issue.

The severity of the disease did not decrease after the intervention, as it was not intended. However, the positive changes occurred in all aspects of QoL of experimental group. This was especially evident in the worry and emotional domains of QoL. It seems that the self-care program has made positive behavioral and cognitive changes in the experimental group. Such behavioral changes were predicted by Johnston and Orem [23,16]. Health-related quality of life generally refers to the patients' perceptions of their physical functioning, social functioning, role functioning, mental health, vitality, pain, and cognitive functioning. In many cases improvements in health-related quality of life are a natural result of improved clinical outcomes. However, patients' perception of their quality of life is also improved when they are empowered by well-designed educational programs. Empowered patients tend to feel more personally capable of positively impacting their outcomes. For patients with chronic conditions, health-related quality of life can improve significantly when they are trained in self-management techniques and empowered with education. Therefore, it could be said that the educational and self-care programs like the programs conducted in the present study could satisfy many needs of these chronic patients and will empower them to improve their quality of life.

### Study limitations

The intervention has two components; the educational sessions, and the subsequent self-care program. However, we do not know exactly to which component the changes in QoL might be attributed. Was it the educational program alone, altering subjects perceptions and understanding, or was it the educational program plus self-care that mediated the change?

Although the active and continual participation of the patients in the management of their own problems may have an important role in the improved QoL in the experimental group, however, how the intervention might be altering QoL as recorded by CLDQ, is the issue that is not completely clear. This is an interesting question for future researches.

Although we tried to use a randomized sample in the experimental and control groups, however, given the small sample size, randomization may not assure a balance between arms.

The CDLQ is the first disease specific HRQL instrument for patients with Chronic Liver Disease. Although the inputs from the patients with a variety of types and stages of liver disease have been used for the development of the scale. However, the number of patients with Childs C were relatively low in the original validation studies [5], so the instrument may have limited validity in patients with Childs C. Yet, we used only the CLDQ for measuring the QoL, however, using other objective scales, such as functional status, etc, could add the reliability of the results in the future studies.

## Conclusion

The result of the present study confirmed the positive effects of the educational and self care programs on the QoL of cirrhotic patients. The excessive aspiration of patients for educational programs was confirmed. Unfortunately no systematic and organized educational program is now existed for these chronic patients. Extensive educational and self-care programs along with long-term follow up such as the program conducted in this study are suggested. There was also some ambiguity related to the component that actually affected the QoL in the present study. So, further study is suggested to know to which component the change in QoL might be attributed.

## Competing interests

The author(s) declare that they no competing interests.

## Authors' contributions

MZ: Initiation and design of the research, collection and analysis of the data and writing the draft paper, MAH: co-analysis and editorial revision of draft papers, MR: supervisor, AKN: supervisor, SMA: supervisor, co-analysis and translation of draft papers,

**Figure 4:**
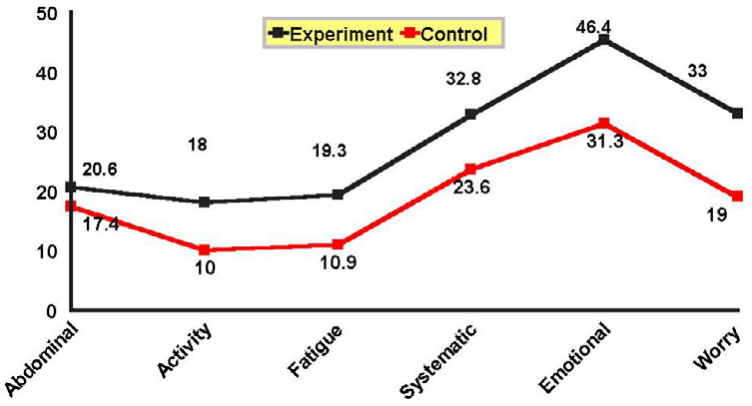
Mean score of CLDQ domains in cirrhotic patients in the experimental and control groups after the intervention

## Supplementary Material

Additional File 1 Self-care in pruritus Click here for file

## References

[B1] Andreoli T (1997). Cecil essential of the medicine.

[B2] Goldberg E, Chopra S Overview of the complications, prognosis and management of cirrhosis. http://Utdol.com/application/topic.asp?file=cirrhosi/9247&type=A&selectedTitle=2~182.

[B3] Alavian SM, Azimi K, Sarafi M, Alavi M, Malek Zadeh R (2002). To determine etiologic factors of liver cirrhosis in hospitalized patients in shariaty hospital. Iranian Journal of Gastroenteria.

[B4] Younossi ZM, Boparai N, Price LL, Kiwi ML, McCormick M, Guyatt G (2001). Health related quality of life in chronic liver disease: the impact of type and severity of disease. Am J Gastroenterol.

[B5] Mihas AA (2001). Cirrhosis of the liver. Postgraduate Medicine.

[B6] Younossi ZM, Guyatt G, Kiwi M, Boparai N, King D (1999). Development of a disease specific questionnaire to measure health related quality of life in patients with chronic liver disease. Gut.

[B7] Younossi ZM, Boparai N, Price LL, Kiwi ML, McCormick M, Guyatt G (2001). Health-related quality of life in chronic liver disease: the impact of type and severity of disease. Am J Gastroenterol.

[B8] Marchesini G, Bianchi G, Amodio P, Salerno F, Merli M, Panella C, Loguercio C, Apolone G, Niero M, Abbiati R (2001). Factors associated with poor health-related quality of life of patients with cirrhosis. Gastroenterology.

[B9] De Bona M, Ponton P, Ermani M, Iemmolo RM, Feltrin A, Boccagni P, Gerunda G, Naccarato R, Rupolo G, Burra P (2000). The impact of liver disease and medical complications on quality of life and psychological distress before and after liver transplantation. J Hepatol.

[B10] Borgaonkar MR, Irvine EJ (2001). Quality of life measurement in gastrointestinal and liver disorders. Gut.

[B11] Davis GL, Balart MD, Schiff ER, Lindsay K, Bodenheimer HC, Perrillo RP, Carey W, Jacobson IM, Payne J, Dienstag JL (1994). Assessing health-related quality of life in chronic hepatitis C using the sickness impact profile. Clin Ther.

[B12] Ware JE, Bayliss MS, Mannocchia M, Davis GL, the International Hepatitis Interventional Therapy Group (1999). Health-related quality of life in chronic hepatitis C: Impact of disease and treatment response. Hepatology.

[B13] Bonkovsky HL, Woolley JM (1999). the Consensus Interferon Study Group. Reduction of health-related quality of life in HCV and improvement with interferon therapy. Hepatology.

[B14] Foster GR, Goldin RD, Thomas HC (1998). Chronic hepatitis C virus infection causes a significant reduction in quality of life in the absence of cirrhosis. Hepatology.

[B15] Younossi ZM, Kiwi ML, Boparai N, Price LL, Guyatt G (2000). Cholestatic liver diseases and health-related quality of life. Am J Gastroenterol.

[B16] Craddock RB, Adams PF, Usui WM, Mitchell L (1999). An intervention to increase use and effectiveness of self-care measures for breast cancer chemotherapy patients. Cancer Nurs.

[B17] Guillemin F, Bombardier C, Beaton D (1993). Cross-cultural adaptation of health-related quality of life measures: literaturereview and proposed guidelines. J Clin Epidemiol.

[B18] Ries AL, Kaplan RM, Limberg TM, Prewitt LM (1995). Effects of Pulmonary Rehabilitation on Physiologic and Psychosocial Outcomes in Patients with Chronic Obstructive Pulmonary Disease. Ann Intern Med.

[B19] Van der Plas SM, Hansen BE, de Boer JB, Stijnen T, Passchier J, de Man RA, Schalm SW (2003). Generic and disease-specific health related quality of life in non-cirrhotic, cirrhotic and transplanted liver patients: a cross-sectional study. BMC Gastroenterol.

[B20] Glis H, Wiklund I (2002). Health-related quality of life and gastrointestinal disease. Journal of Gastroenterology and Hepatology.

[B21] Bernstein D Health Related Quality of Life and Hepatitis C. http://www.hcvadvocate.org/hcsp/articles/Bernstein-2.html.

[B22] Gralnek IM, Hays RD, Kilbourne A, Rosen HR, Keeffe EB, Artinian L, Kim S, Lazarovici D, Jensen DM, Busuttil RW, Martin P (2000). Development and evaluation of the Liver Disease Quality of Life instrument in persons with advanced, chronic liver disease – the LDQOL 1.0. Am J Gastroenterol.

[B23] Johnston M (1999). Mood in chronic disease: Questioning the answer. Curr Psychol.

