# Supplementary material for: Effects of a self-care program on quality of life of cirrhotic patients referring to Tehran Hepatitis Center
Source: Health Qual Life Outcomes. 2005 May 18;3:35. doi: 10.1186/1477-7525-3-35 (PMC1156929; doi:10.1186/1477-7525-3-35)
Supplement: Additional File 1 — Self-care in pruritus [file 1477-7525-3-35-S1.doc]

| Fourth Week Third Week Second Week First Week | | | | | | | | | | | | | | | | | | | | | | | | | | | | Date |
| --- | --- | --- | --- | --- | --- | --- | --- | --- | --- | --- | --- | --- | --- | --- | --- | --- | --- | --- | --- | --- | --- | --- | --- | --- | --- | --- | --- | --- |
| 7 | 6 | 5 | 4 | 3 | 2 | 1 | 7 | 6 | 5 | 4 | 3 | 2 | 1 | 7 | 6 | 5 | 4 | 3 | 2 | 1 | 7 | 6 | 5 | 4 | 3 | 2 | 1 | Consideration |
|  |  |  |  |  |  |  |  |  |  |  |  |  |  |  |  |  |  |  |  |  |  |  |  |  |  |  |  | Avoiding warm moist condition |
|  |  |  |  |  |  |  |  |  |  |  |  |  |  |  |  |  |  |  |  |  |  |  |  |  |  |  |  | Wearing light clothes |
|  |  |  |  |  |  |  |  |  |  |  |  |  |  |  |  |  |  |  |  |  |  |  |  |  |  |  |  | Keeping the environment as cool as possible |
|  |  |  |  |  |  |  |  |  |  |  |  |  |  |  |  |  |  |  |  |  |  |  |  |  |  |  |  | Keeping the bedroom cool |
|  |  |  |  |  |  |  |  |  |  |  |  |  |  |  |  |  |  |  |  |  |  |  |  |  |  |  |  | Taking a shower before going to sleep |
|  |  |  |  |  |  |  |  |  |  |  |  |  |  |  |  |  |  |  |  |  |  |  |  |  |  |  |  | Avoid of using a lot of soap & other detergents |
|  |  |  |  |  |  |  |  |  |  |  |  |  |  |  |  |  |  |  |  |  |  |  |  |  |  |  |  | Avoid of using pruritogens ( tomato, meat, egeplant, ...) |
|  |  |  |  |  |  |  |  |  |  |  |  |  |  |  |  |  |  |  |  |  |  |  |  |  |  |  |  | Keeping the nail short |
|  |  |  |  |  |  |  |  |  |  |  |  |  |  |  |  |  |  |  |  |  |  |  |  |  |  |  |  | Mild compressing instead of scraching the itching areas |
|  |  |  |  |  |  |  |  |  |  |  |  |  |  |  |  |  |  |  |  |  |  |  |  |  |  |  |  | Don’t use of antipruriitic drugs with out doctor perscribed |
|  |  |  |  |  |  |  |  |  |  |  |  |  |  |  |  |  |  |  |  |  |  |  |  |  |  |  |  | Daily assessment of skin integrity |
|  |  |  |  |  |  |  |  |  |  |  |  |  |  |  |  |  |  |  |  |  |  |  |  |  |  |  |  | Ontime application of topical antipruritus ointment |
|  |  |  |  |  |  |  |  |  |  |  |  |  |  |  |  |  |  |  |  |  |  |  |  |  |  |  |  | Avoid of taking a hot bath or shower |
|  |  |  |  |  |  |  |  |  |  |  |  |  |  |  |  |  |  |  |  |  |  |  |  |  |  |  |  | Using light bedclothes |

***Self- care in pruritus***
